# Effects of a Personalized Augmented Reality Exercise Program Based on Basic Fitness on Key Components of Physical Fitness in Healthy Adults: A Randomized Controlled Trial

**DOI:** 10.3390/bioengineering12121354

**Published:** 2025-12-12

**Authors:** Jaewon Lee, Jaeho Yu

**Affiliations:** Department of Physical Therapy, Sunmoon University, Asan 31460, Republic of Korea; zzxvv99@gmail.com

**Keywords:** augmented reality, physical fitness, physical factors, multicomponent training program

## Abstract

Background: Augmented reality (AR)-based exercise offers a low-cost option for home training, but the lack of supervision increases the risk of incorrect performance. Therefore, AR systems must demonstrate accuracy and effectiveness comparable to therapist-led training. To address this need, this study examined whether an AR-supported multi-component exercise program improves six key components of physical fitness. Methods: Twenty-eight healthy adults from South Korea were recruited and randomly assigned to either the AR group or the physical therapist (PT) group. Participants were assessed for six components of physical fitness: muscular strength, muscular endurance, balance, cardiorespiratory endurance, flexibility, and coordination. Each group performed the exercise program three times per week for four weeks, with each session lasting at least 30 min. Statistical analyses were conducted using paired *t*-tests to evaluate pre- and post-intervention effects within each group and independent *t*-tests to compare differences between the two groups. The level of statistical significance was set at *p* < 0.05. Results: Exploratory analyses showed that both groups demonstrated post-intervention improvements in muscular endurance, balance, flexibility, and coordination, while cardiorespiratory endurance and grip strength showed no notable changes. Between-group comparisons revealed significant differences only in right-hand relative grip strength at both baseline and post-intervention, with no other domains differing between groups. Conclusions: First, the AR-based intervention significantly improved muscular endurance, balance, flexibility, and coordination. Second, there were no significant differences between the AR-based and therapist-led interventions. Consequently, AR-based exercise programs may enhance specific components of physical fitness and could be effectively utilized to complement and extend therapist-led training sessions.

## 1. Introduction

Exercise and rehabilitation are sometimes restricted by environmental factors such as weather, location, and transportation. Advances in science and technology have minimized the impact of these limitations, and particularly, virtual and augmented reality (AR) technologies have emerged as novel approaches for promoting health [1]. AR-based rehabilitation environments go beyond conventional exercise methods by providing users with new and engaging experiences, which may increase their motivation and adherence to physical training. During this process, physical therapists can analyze exercise data to objectively evaluate users’ physical outcomes and adjust exercise plans accordingly [2]. The expansion of AR-based exercise sessions also enables individuals to engage in physical training at home at a relatively low cost [3]. However, home-based exercises without professional supervision make immediate correction difficult and may increase the risk of performing movements incorrectly [4]. To overcome these challenges, ongoing research has focused on encouraging user participation while maintaining accuracy and efficiency comparable to traditional supervised exercise systems [5].

According to previous studies, various forms of feedback—such as auditory, visual, textual, and video-based feedback—have been shown to enhance user motivation and facilitate not only physical activity but also cognitive learning [5]. In particular, auditory feedback demonstrated superior effectiveness in guiding users toward accurate movements [5]. Moreover, an analysis of nine AR systems incorporating different types of feedback for shoulder rehabilitation revealed clear advantages over conventional rehabilitation methods in terms of usability, enjoyment, motivation, and exercise performance outcomes [6]. However, this does not imply that traditional exercise or rehabilitation methods are ineffective. Exercise programs that include strength and flexibility training performed at appropriate intensities and frequencies are effective in delaying neuromuscular capacity decline, preventing or reducing sarcopenia, and minimizing age-related changes in body composition [7]. Therefore, combining augmented reality technology with traditional exercise methods may yield more effective outcomes.

Maintaining good health is essential, and as aging progresses, physical fitness and self-efficacy play a crucial role in improving quality of life and overall well-being. Therefore, preventive exercise interventions should be initiated before the onset of disease to promote health [8]. Physical fitness comprises multiple components: (1) muscular components such as strength and endurance, (2) motor components such as balance, (3) cardiorespiratory components, (4) metabolic components, and (5) morphological components such as height and subcutaneous fat [9]. These elements are closely associated with an individual’s health status and level of physical activity. Accordingly, exercise programs should be systematically designed to promote these components of physical fitness for the maintenance and enhancement of overall health.

Multi-component exercise is a comprehensive intervention method that combines several elements of physical fitness, such as muscular strength, flexibility, and cardiorespiratory endurance. Compared to conventional single-component exercise programs, it has been shown to exert more positive effects on fitness outcomes and plays a key role in improving quality of life and preventing falls [7,10]. In a study involving older adults residing in long-term care facilities, progressive multi-component exercise was proven effective in preventing declines in cardiopulmonary health [11]. Advances in science and technology have further enhanced the implementation of such exercises by providing effective tools to facilitate them. Exercise programs in the form of video games—commonly referred to as exergames—that include strength and balance training have been found to provide greater motivation to users and contribute positively to both physical and cognitive function [12].

Previous studies have demonstrated that multi-component exercise can produce various physiological benefits that contribute to improved physical fitness. In particular, continuous research is needed to verify the effectiveness of AR-based rehabilitation environments in order to expand and enhance therapeutic sessions. Therefore, the purpose of this study was to investigate the effects of an AR-based multi-component exercise program on six components of physical fitness—muscular strength, muscular endurance, balance, cardiorespiratory endurance, flexibility, and coordination—and to test the hypothesis that this intervention would be effective in improving these components.

## 2. Materials and Methods

### 2.1. Study Design

Participants were recruited through public advertisements and were screened for eligibility by the principal investigator A according to predefined inclusion criteria. Randomization was performed by an independent assistant using a simple randomization procedure based on the RANDBETWEEN function in Microsoft Excel, which generated random allocation codes assigning participants to either the augmented reality group (ARG) or the physical therapist-led group (PTG). Allocation was concealed from investigator A; group assignments were disclosed only after each participant arrived for the first visit, at which point the assistant directed the participant to the assigned intervention room. The study took place at SUNMOON University in Asan-Si, South Korea, within the Health and Medical Sciences Building. The ARG intervention was delivered in Room 104 and the PTG intervention in Room 114, each administered by separate interventionists (C and D). Because recruitment materials described the study only as a physical fitness enhancement program and interventions were conducted in separate rooms by different interventionists, participants were unaware of their group assignment and had minimal opportunity to encounter members of the other group. All outcomes were assessed by assessor B, who remained blinded to group allocation throughout the study. This study was approved by the Institutional Review Board of Sunmoon University (IRB No. SM-202206-030-2) and was prospectively registered in the Clinical Research Information Service (CRIS; KCT0008368).

### 2.2. Participants

A total of 28 healthy adults were enrolled in this exploratory randomized controlled trial, which was conducted from October 2022 to February 2023. Although a formal confirmatory power calculation was not required for this exploratory design, we conducted a preliminary feasibility check using G*Power (version 3.1.9.7) [13] to ensure that the planned sample size was reasonable for detecting meaningful changes in key outcomes. Based on this assessment and practical considerations for feasibility, a target sample size of approximately 24–28 participants were deemed appropriate, and 28 participants were ultimately recruited. After eligibility screening based on predefined inclusion and exclusion criteria, all 28 individuals were randomized, and all completed the 4-week intervention without loss to follow-up or protocol deviations.

Participants were eligible if they met the following criteria: (1) no history of musculoskeletal surgery, trauma, or neurological disorders within the past six months; (2) no cardiovascular or pulmonary diseases; (3) absence of pain that could restrict exercise; (4) no psychiatric or cognitive disorders within the past two years; and (5) no vestibular dysfunction. Participants who experienced discomfort during the intervention were allowed to rest; those unable to continue due to COVID-19 infection were excluded. All participants provided written informed consent before enrollment.

Baseline height was measured using an automatic BMI measuring instrument (BSM 370, Republic of Korea, 2011), and weight was measured using a body composition analyzer (InBody 570, Biospace, Republic of Korea, 2013). Physical characteristics are summarized in Table 1.

### 2.3. Experiment Procedures

**Figure 1:**
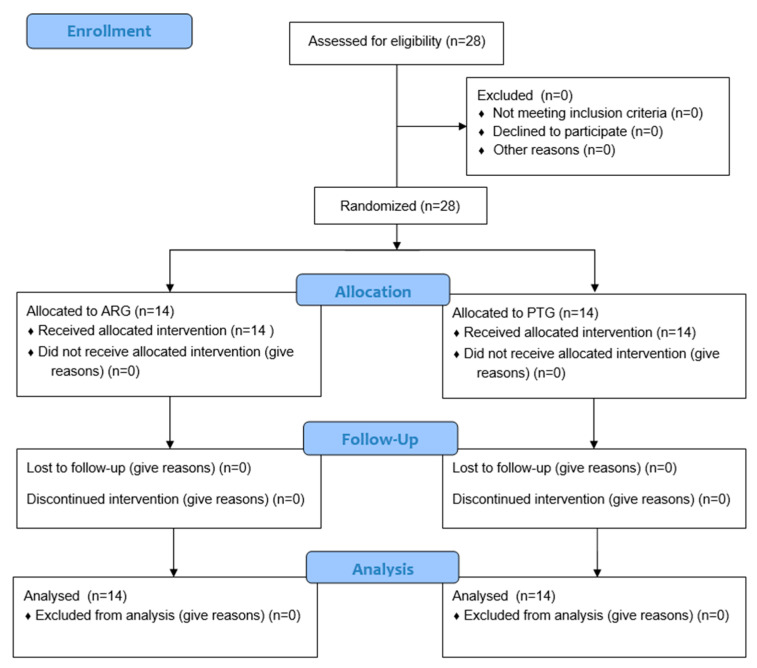
Consort Flowchart.

The study procedure is illustrated in [Figure 1]. Participants underwent baseline assessments followed by randomization to either the ARG or PTG. All participants were evaluated twice—before and after the intervention—for six physical fitness components: muscular strength, muscular endurance, balance, cardiorespiratory endurance, flexibility, and coordination.

The exercise program was conducted three times per week for four weeks, totaling 12 sessions, with each session lasting 30 min. Stretching exercises were performed for three sets, maintaining each position for 15 s, while each strengthening exercise was performed for three sets of 12 repetitions. The intensity of the exercise tools used during the sessions was progressively increased each week to correspond to a Borg Rating of Perceived Exertion of 13–15.

### 2.4. Measurement Tools and Methods

All participants were measured for height and weight once before the intervention. Assessments of muscular strength, muscular endurance, balance, cardiorespiratory endurance, flexibility, and coordination were conducted twice—once before and once after the intervention. To minimize measurement error, all assessments were performed by the same researcher using standardized procedures and testing environments.

#### 2.4.1. Measuring Strength

Muscular strength was assessed using a handgrip dynamometer (Jamar^®^, Preston, CT, USA). Participants were seated comfortably in a chair with the test arm slightly abducted at approximately 15° from the trunk and the elbow fully extended, ensuring that the arm did not touch the body. Following the researcher’s verbal instruction, participants were asked to maintain the position and exert maximal grip force for 3 s. The researcher then recorded the value. Handgrip strength was measured twice alternately for both the right and left hands, and the highest value was recorded to the nearest 0.1 kg. Relative grip strength was calculated by dividing the measured grip strength by the participant’s weight [14].

#### 2.4.2. Measuring Muscular Endurance

Muscular endurance was assessed using the 30-s chair stand test (30CST), performed on a chair with a backrest but no armrests. Participants sat in the middle of the chair with their back straight, feet flat on the floor, and arms crossed at the wrists and placed across the chest. Upon the researcher’s signal, participants stood up fully and then returned to a seated position. To ensure proper form, each participant practiced one or two times before the test. They were then instructed to perform as many full stands as possible within 30 s. The total number of completed sit-to-stand cycles within the 30-s period was recorded [15].

#### 2.4.3. Measuring Balance

Balance was evaluated using the Timed Up and Go (TUG) test. One cone and one chair with a backrest but no armrests were prepared, positioned to face each other. The cone was placed exactly 3 m away from the front edge of the chair, ensuring a straight line between the two. Participants sat upright in the middle of the chair with their back straight, feet flat on the floor, and hands resting on their thighs. One foot was placed slightly ahead of the other, with the trunk leaning slightly forward. At the researcher’s signal, participants stood up, walked as quickly as possible around the cone, and returned to sit on the chair. After one practice trial, participants performed the test twice, and the fastest time was recorded to the nearest 0.1 s [16].

#### 2.4.4. Measuring Cardiopulmonary Endurance

Cardiorespiratory endurance was assessed using the 2-Minute Step Test (2MST). Upon the examiner’s signal, participants began stepping in place, starting with the right foot. One full step was counted when both feet alternately touched the ground. The target knee height for each participant was individually determined by measuring the distance from the midpoint of the patella to the anterior superior iliac spine using a tape measure; participants were instructed to raise their knees to at least the midpoint level of the thigh. If the knee height was not maintained, participants were reminded to correct their posture, but the step was not counted while the timer continued. Participants were instructed to perform as many steps as possible within the 2-min period [17].

#### 2.4.5. Measuring Flexibility

Flexibility was assessed using the Sit-and-Reach Test (SRT). Participants removed their shoes and sat upright with their knees fully extended and the soles of their feet placed flat against the vertical surface of the measurement box. The distance between the feet was kept within 5 cm. With both arms extended forward, participants reached as far as possible while keeping their knees straight and avoiding any bouncing or jerking motion. The fingertips of both hands were required to remain aligned throughout the movement. The test was performed twice, and the better score was recorded to the nearest 0.1 cm [18].

#### 2.4.6. Measuring Coordination

Coordination was evaluated using the Figure 8 walking test (F8W). A rectangular course measuring 3.6 m in length and 1.6 m in width was marked on the laboratory floor, with cones placed at both rear corners and a chair positioned 2.4 m from each cone. Participants sat on the front center edge of the chair and began the test upon the examiner’s signal. They stood up, walked around the cone positioned diagonally to the right, returned to sit down, then immediately stood up again and walked around the cone diagonally to the left before sitting back down. The total time required to complete the task was recorded to the nearest 0.1 s. No practice trials were provided, and the use of any assistive devices was not permitted [19].

### 2.5. Intervention Method

**Figure 2:**
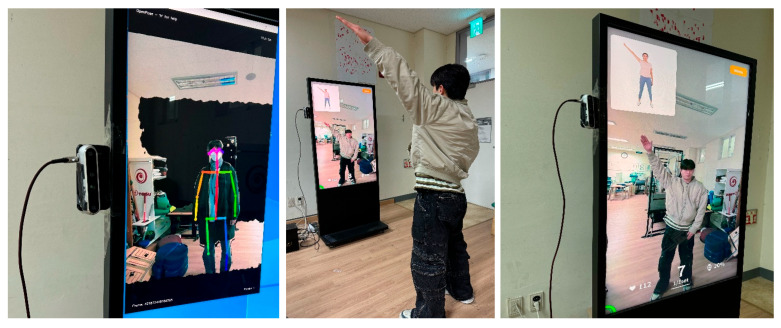
Augmented reality-based intervention method.

Participants in the ARG performed the exercises using an AR-device [Figure 2]. A markerless RGB camera was used to project each participant’s body onto the screen and identify anatomical landmarks such as joint positions. The physical fitness assessment was delivered in a fixed sequence ranging from muscle strength to coordination, accompanied by guided videos and on-screen text to ensure that even first-time users could easily follow the instructions. More than 500 stored exercises were categorized by difficulty level (high, medium, low) and by target joints or functional components. Using cut-off values determined according to the age-specific grading standards of Korea’s National Fitness Award program, the system automatically assigned the appropriate difficulty level and generated an exercise program tailored to each participant’s assessment results [20]. The therapist then established training goals across six components, and a unified protocol was developed for all participants as presented in Table 2. The exercise program and prescribed repetitions generated by the ARG system were applied identically to the PTG, while training intensity was individually adjusted according to assessment outcomes and the therapist’s clinical judgment. During the exercises, the system provided audiovisual feedback and recorded accuracy scores in real time.

Participants in the PTG performed the exercises under the supervision of a therapist using traditional one-on-one methods. Before the intervention, the researcher explained the entire procedure and exercise program to all participants and recommended wearing comfortable clothing to ensure accurate and safe performance. The experimental environments for the two groups were assigned separately to prevent overlap, and the researcher continuously monitored both settings to maintain consistent and safe conditions.

### 2.6. Data Analysis

Descriptive statistics were used to calculate the general characteristics of the participants, and the mean and standard deviation were computed for all variables. All statistical analyses were performed using IBM SPSS Statistics version 26.0 (IBM Corp., Armonk, NY, USA). To evaluate the effects of the exercise program before and after the intervention within each group, paired *t*-tests were conducted, and independent *t*-tests were used to compare differences between the two groups. The level of statistical significance was set at α = 0.05.

## 3. Results

**Figure 3:**
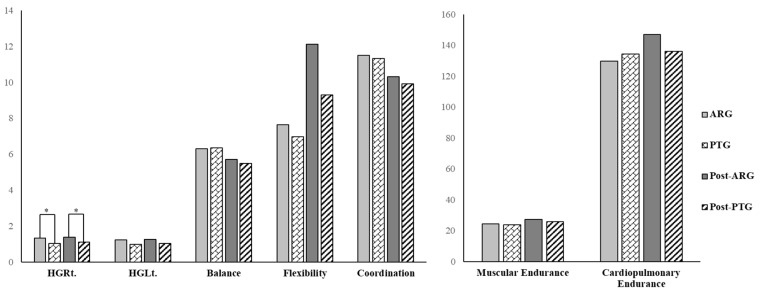
Comparison between groups after intervention; * *p* < 0.05, HGRt: hand grip right side, HGLt: hand grip left side, ARG: augmented reality exercise group, PTG: physical therapy exercise group.

Normality assumptions were confirmed prior to analysis using the Shapiro–Wilk test. At baseline, a significant difference was observed between the two groups in right-hand relative grip strength (*p* < 0.05), while no significant differences were found in any other variables (*p* > 0.05) [Figure 3, Table 3].

**Figure 4:**
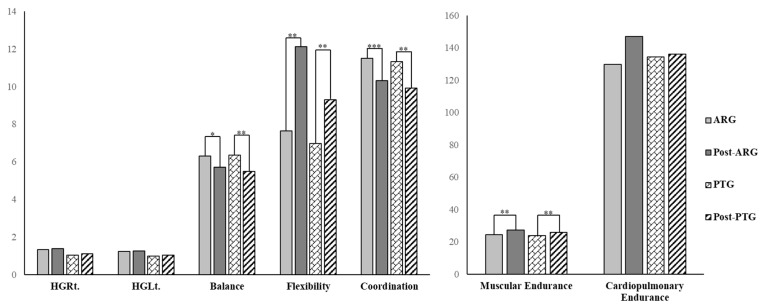
Comparison within a group after intervention.; * *p* < 0.05, ** *p* < 0.01, *** *p* < 0.001, HGRt: hand grip right side, HGLt: hand grip left side, ARG: augmented reality exercise group, PTG: physical therapy exercise group.

After the intervention, no significant differences were observed in relative grip strength (muscular strength) for either hand in both groups (*p* > 0.05). Muscular endurance significantly improved in both groups (*p* < 0.01). Balance showed significant improvement in both the ARG (*p* < 0.05) and the PTG (*p* < 0.01). No significant changes were found in cardiorespiratory endurance in either group (*p* > 0.05). Flexibility significantly improved in both groups (*p* < 0.01). Coordination also improved significantly in the ARG (*p* < 0.001) and in the PTG (*p* < 0.01) [Figure 4, Table 4].

After the intervention, a significant difference between groups was found in right-hand relative grip strength (*p* < 0.05), while no other variables showed significant between-group differences (*p* > 0.05) [Figure 3, Table 3].

## 4. Discussion

Many studies have reported that low muscle function is associated with higher mortality and morbidity rates [21]. Handgrip strength is widely used as a surrogate measure of overall muscle strength because it is simple, cost-effective, and easy to administer [22]. It has shown a strong association with the incidence and mortality of various diseases—including cardiovascular disease, respiratory disease, chronic obstructive pulmonary disease and cancer—and weaker grip strength is linked to poorer health outcomes, making it a reliable prognostic indicator [21,23]. Moreover, the predictive ability of grip strength for mortality remains consistent regardless of whether it is expressed in absolute or relative terms [24]. In the present study, although grip strength increased in both hands after the intervention in both groups, the improvement was not statistically significant. This finding may be explained by the similarity between the evaluated task and the type of exercise performed; greater effects are typically observed when the exercise closely matches the test variable. It has been recommended that to increase grip strength specifically, individuals should train at approximately 75% of one-repetition maximum for at least nine weeks [25]. However, studies reporting large effects often adopt a multi-component exercise approach, suggesting that integrating balance, flexibility, and endurance training may yield additional benefits for grip strength improvement [26]. Therefore, continued participation in this program or the addition of grip strength–specific exercises after reassessment may further enhance results. Additionally, this study did not stratify participants based on handedness or sex, as the primary goal was to verify the effectiveness of the AR system and the exercise protocol. Since most participants were right-handed, the authors speculate that this may have contributed to the pre- and post-intervention differences observed between groups.

Exercises included in this intervention, such as ankle sandbag kicks and step training, were sufficient to improve muscular endurance. A previous 5-week AR-based exergame intervention for community-dwelling older adults significantly enhanced lower-limb strength and functional balance performance, consistent with the findings of the present study [27]. Because lower-limb function is closely associated with balance, the 30CST is often used not only to evaluate lower-limb strength and muscular endurance but also to assess balance ability and predict fall risk [28,29]. Furthermore, when an augmented reality–based Otago exercise program was applied to older adults at high risk of falls residing in long-term care facilities, the AR group showed greater median improvements in both the Berg Balance Scale and 30CST scores compared with the conventional walking group [30].

As mentioned earlier, lower-limb function is closely related to balance. Groups with reduced lower-limb function, such as those experiencing sarcopenia and associated muscle loss, have demonstrated poorer performance on the TUG test [31,32]. A recent meta-analysis reported that AR–based exercise interventions effectively improved balance and gait ability, showing particularly strong effects in elderly populations with neurological disorders such as stroke and Parkinson’s disease [33]. This may be attributed to the fact that older adults typically rely heavily on visual information; thus, AR-based exercise enhances proprioceptive feedback in the lower limbs, leading to improved balance control. These findings are consistent with the results of the present study [34,35].

Modern individuals who spend prolonged periods sitting are particularly susceptible to excessive strain in the hamstring muscles [36]. The SRT has been shown to provide a valid and reliable measure for assessing hamstring flexibility [18], and the calf muscles are also known to influence SRT performance [37]. In this study, static stretching interventions targeting the hamstring and calf muscles were effective, consistent with findings from previous research [38]. Furthermore, a study involving 68 healthy adults demonstrated that remote rehabilitation performed four times per week for eight weeks produced significantly greater improvements in flexibility compared with a conventional home exercise group [39]. Similarly, the results of the present study align with previous findings showing that both a 4-week AR-based proprioceptive exercise program and a 12-week AR-based sarcopenia prevention program significantly improved hamstring flexibility [34,40].

The 2MST is one of the commonly used assessments for cardiorespiratory endurance, similar to the 6-Minute Walk Test. It is more practical in limited physical spaces and allows rapid evaluation of aerobic capacity [41,42]. However, several studies have demonstrated that the 2MST is also correlated with lower-limb strength and muscular endurance, as well as with dynamic balance measured by the TUG test, indicating its close association with functional mobility [42,43,44]. Previous research has shown that AR-based exercise interventions improved cardiorespiratory endurance in older adults with reduced aerobic capacity [45]. In contrast, the present study found no statistically significant improvement in cardiorespiratory endurance, despite significant changes in other variables. This may be due to insufficient exercise duration. According to the American College of Sports Medicine (ACSM) guidelines, only sessions lasting at least 10 min can be accumulated toward total exercise time, although bouts shorter than 10 min may still provide beneficial adaptations when performed as part of high-intensity aerobic training [46]. Therefore, it may be necessary to increase the proportion and duration of aerobic components in future exercise protocols.

The F8W requires participants to perform both straight and curved paths, including clockwise and counterclockwise turns. Thus, individuals must integrate multiple sensory inputs and plan goal-directed movements to navigate complex spatial configurations. This test also demands coordination of lower-limb function and cognitive processing to smoothly transition between movement patterns [47]. AR interfaces provide significant visual advantages by facilitating the relationship between visual attention and the brain’s visual information processing [48]. In this study, projecting the user’s image onto the screen offered visual feedback necessary for interaction, while guided exercise videos supported initial motor learning for beginners [6]. Previous studies have shown that similar AR interfaces effectively alleviated phantom limb pain in amputees [49,50]. According to social cognitive theory, which emphasizes individual learning through continuous interaction with the environment, behavior is fundamentally learned and influenced by two key cognitive factors—self-efficacy and outcome expectancy—that determine behavioral adherence [51]. Self-efficacy plays a crucial role in promoting participation in physical activity, and AR-based environments provide strong intrinsic motivation by enhancing users’ sense of agency and engagement [52].

This study has several limitations. First, participants’ daily activities outside the experimental environment could not be fully controlled, potentially influencing the outcomes. Second, because the exercise program was individualized based on baseline assessments, some variables may not have shown significant changes. Third, as the participants were healthy adults in their twenties, the generalizability of the findings to other age groups or clinical populations is limited. Although previous studies have reported that AR-based exercise interventions can improve cognitive and physical functions across diverse age groups and clinical conditions—such as stroke, Parkinson’s disease, and dementia—future studies should include broader age ranges and clinical samples and incorporate long-term follow-up assessments to evaluate sustained effects. Finally, despite careful planning, the relatively small sample size (14 participants per group) means that the study was underpowered to rule out modest between-group differences, particularly in the presence of baseline imbalance. Therefore, nonsignificant between-group results should be interpreted with caution and validated in larger, adequately powered trials.

## 5. Conclusions

This study aimed to investigate the effects of an AR-based multi-component exercise program on six physical fitness factors—muscular strength, muscular endurance, balance, cardiorespiratory endurance, flexibility, and coordination. The findings were as follows: First, the AR-based intervention significantly improved muscular endurance, balance, flexibility, and coordination. Second, the magnitude of improvement observed in the AR group was generally comparable to that of the therapist-led group, although no statistically significant between-group differences were detected. Given the pilot nature and limited statistical power of this study, these non-significant differences should not be interpreted as evidence of equivalence or noninferiority between the two interventions. In conclusion, AR-based exercise programs show promise for enhancing specific components of physical fitness and may serve as a supplementary modality to therapist-led sessions; however, larger and adequately powered trials are required to confirm comparative effectiveness.

## Figures and Tables

**Table 1:** General characteristics of participants.

	ARG (*n* = 14)	PTG (*n* = 14)
Age (years)	22.29 ± 1.68	22.86 ± 3.01
Height (cm)	169.33 ± 8.18	162.66 ± 5.96
Weight (kg)	70.61 ± 16.08	59.33 ± 10.76

Values indicate mean ± standard deviation, ARG: augmented reality exercise group, PTG: physical therapist exercise group.

**Table 2:** Exercise program.

Name of the Exercise	Explanation of Exercise
Standing shoulder extension	The subjects stand up straight with their shoulders back and their legs spread shoulder width apart. They place their hands behind their back with their palms facing backward and interlace their fingers. Then, they slowly lift their arms up and hold the position (15 s holding × 3 sets).
Standing dumbbell bicep curls with one arm bent	Stand up straight with your back straight and your feet shoulder-width apart. Keep your arms close to your sides with your palms facing forward and grab the dumbbells. Bend your elbows and lift the dumbbells up. Return to the starting position and repeat (12 times × 3 sets).
Standing forward bend	Stand up straight with your back straight and your arms at your sides. Slowly bend your head and torso while keeping your arms close to your sides until your fingertips touch the ground. Then return to the starting position (15 s holding × 3 sets).
One-legged Medicine Ball Diagonal Lift	Stand with a straight back and grab a medicine ball with both hands. Balance on one leg and bend the hip and knee joint on the supporting side, lowering the medicine ball in a diagonal direction. Straighten the supporting leg and simultaneously lift the medicine ball upwards towards the opposite diagonal direction, bending the hip and knee joint on the same side (12 times × 3 sets × each side)
Sandbag Ankle Steps	Put sandbags on your ankles and stand comfortably. Then, lift your knee up to the middle of your thigh and step up and down with each foot, as if climbing stairs (2 min × 3 sets).
Stand on one foot on a step box and lift your knee up	Stand on a step box with one leg and bend the hip and knee joints of the other leg while stepping up onto the box. Then return to the starting position and repeat with the other leg (12 times × 3 sets × each side)
Standing Calf Stretch with knee extension	Stand with your feet placed apart. Maintain the heel of the rear foot not to fall, then slowly bend the back knee and maintain the feeling of pulling the calf muscle of the rear foot (15 s holding × 3 sets)

**Table 3:** Descriptive Statistics and Between-Group Comparisons at Pre- and Post-Intervention.

		Pre			Post		
		ARG	PTG	t	ARG	PTG	t
**Strength**							
**Hand grip**	**Rt**	1.34 ± 0.42	1.02 ± 0.21	2.556 *	1.38 ± 0.41	1.10 ± 0.25	2.198 *
	**Lt**	1.24 ± 0.45	0.97 ± 0.26	1.946	1.25 ± 0.40	1.02 ± 0.25	1.825
***Muscular Endurance***		24.43 ± 6.87	23.71 ± 4.76	0.320	27.29 ± 7.10	26.00 ± 5.35	0.541
***Balance***		6.30 ± 0.95	6.35 ± 1.12	−0.106	5.72 ± 0.82	5.49 ± 0.68	0.826
**Cardiopulmonary Endurance**		129.79 ± 29.10	134.29 ± 18.17	−0.491	146.93 ± 43.59	136.00 ± 24.06	0.821
***Flexibility***		7.63 ± 11.15	6.98 ± 9.34	0.167	12.11 ± 8.77	9.30 ± 9.39	0.816
***Coordination***		11.49 ± 2.11	11.33 ± 1.88	0.206	10.31 ± 1.49	9.91 ± 1.12	0.802

* *p* < 0.05, mean ± standard deviation, Rt: right, Lt: left, ARG: augmented reality exercise group, PTG: physical therapy exercise group.

**Table 4:** Comparison within a group after intervention.

		ARG			PTG		
		Pre	Post	t	Pre	Post	t
**Strength**							
**Hand grip**	**Rt**	1.34 ± 0.42	1.38 ± 0.41	−0.843	1.02 ± 0.21	1.10 ± 0.25	−1.693
	**Lt**	1.24 ± 0.45	1.25 ± 0.40	−0.144	0.97 ± 0.26	1.02 ± 0.25	−1.105
***Muscular Endurance***		24.43 ± 6.87	27.29 ± 7.10	−3.899 **	23.71 ± 4.76	26.00 ± 5.35	−3.829 **
***Balance***		6.30 ± 0.95	5.72 ± 0.82	2.891 *	6.35 ± 1.12	5.49 ± 0.68	3.770 **
**Cardiopulmonary Endurance**		129.79 ± 29.10	146.93 ± 43.59	−1.864	134.29 ± 18.17	136.00 ± 24.06	−0.265
***Flexibility***		7.63 ± 11.15	12.11 ± 8.77	−3.898 **	6.98 ± 9.34	9.30 ± 9.39	−3.872 **
***Coordination***		11.49 ± 2.11	10.31 ± 1.49	4.474 ***	11.33 ± 1.88	9.91 ± 1.12	3.887 **

* *p* < 0.05, ** *p* < 0.01, *** *p* < 0.001, mean ± standard deviation, Rt: right, Lt: left, ARG: augmented reality exercise group, PTG: physical therapy exercise group.

## Data Availability

The datasets generated and analyzed during the current study are not publicly available due to institutional policy but are available from the corresponding authors on reasonable request.

## Abbreviations

AR	Augmented reality
ARG	Augmented reality exercise group
PT	Physical therapist
PTG	Physical therapist exercise group
30CST	30-second chair stand test
TUG	Timed Up and Go
2MST	2-Minute Step Test
SRT	Sit-and-Reach Test
F8W	Figure 8 walking test
HGRt	Hand grip right side
HGLt	Hand grip left side
